# Pinless Navigation in Unicompartmental Knee Arthroplasty

**DOI:** 10.3390/jcm10112422

**Published:** 2021-05-30

**Authors:** Sarah Keuntje-Perka, Philipp von Roth, Michael Worlicek, Matthias Koch, Volker Alt, Moritz Kaiser

**Affiliations:** 1 Sporthopaedicum Straubing, Medical Care Center, 94135 Straubing, Germany; sarahperka@googlemail.com (S.K.-P.); roth@sporthopaedicum.de (P.v.R.); moritz.kaiser@ukr.de (M.K.); 2 Department of Trauma Surgery, Regensburg University Medical Center, 93053 Regensburg, Germany; matthias.koch@ukr.de (M.K.); volker.alt@ukr.de (V.A.)

**Keywords:** knee arthroplasty, unicompartmental, navigation, pinless

## Abstract

Purpose: In contrast to total knee arthroplasty (TKA), unicompartmental knee arthroplasty (UKA) is a true resurfacing procedure, as none of the ligaments are replaced or released, and the pre-arthritic leg alignment is the major goal. As such, the alignment of the tibial component plays a crucial role in postoperative knee function and long-term survival. Pinless navigation has shown reliable results in total knee arthroplasty. To the best of our knowledge, the use of pinless navigation has not been investigated for UKA. Therefore, the present study investigated whether implantation of the tibial component in 3° varus, which is closer to the anatomical axis, is feasible with a pinless optical navigation system. Methods: 60 patients with the diagnosis of an unicompartmental arthritis, were eligible for UKA and treated with implantation in 3° varus alignment of the tibial component. Two groups were established. In the treatment group the tibial component was aligned using a pinless navigation technique. In the control group, a conventional extramedullary alignment guide was used. A clinical and radiographic follow up took place within 1 year of operation. Results: 57 Patients were eligible for analysis. No clinical incidents were noted in the follow up period. The desired target value, the position of the tibial component, was accurately achieved with an average of 3° medial inclination using the pinless navigation as well as using the conventional technique. Mean incision to suture time was negligible between the two techniques. The mean suture time was 43.2 min with pinless navigation and 42.7 min with the conventional technique. Conclusions: With pinless navigation in UKA, a method was presented that made it possible to achieve sled prosthesis alignment at the level of a high-volume surgeon. These results were achieved with an irrelevant increase of surgical time and without placement of pins.

## 1. Introduction

Currently, there is an increasing interest in unicondylar knee arthroplasty (UKA). Compared to total knee arthroplasty (TKA), there are fewer postoperative complications such as wound alterations or periprosthetic infections [1,2]. In some studies it could also be shown that the early outcome, especially with regard to function, is better than with TKA [2,3].

However, UKA seems to have a higher long-term revision rate compared to TKA [1,2]. The reasons for this are quite unclear, as there are almost no studies that investigated to optimal implant position in UKA.

While UKA is the only so-called true surface replacement, since both cruciate ligaments are preserved and the aim of the postoperative alignment is the pre-arthritic status, the accuracy of implantation is of high relevance for the long-term success.

Up to now, the tibial component has been implanted at a 90° angle with respect to the coronal longitudinal axis, but the native knee joint line has approximately 3° varus. Only one study investigated slightly varus-aligned tibia implants in UKA and reported a longer survival rate than neutral alignment [4].

It has also been shown that implantation accuracy and clinical outcome can be improved by using robotic-assisted and computer-navigated systems using pins in TKA and UKA [5,6], but there is a lack of research for pinless navigation in UKA. In UKA, navigation with pins is not appropriate, as the procedure would become unnecessarily invasive due to an extended surgical field.

In this prospective, randomized and controlled single-center trial we investigated whether pinless navigation in UKA is suitable and whether implantation accuracy of the tibial implant can be increased by using a pinless navigation system, compared to conventional alignment with an extramedullary alignment rod. The main target value was the tibial implant in an alignment with a 3° varus.

The hypothesis was that pinless navigation in UKA can provide alignment comparable to that achieved by a high-volume surgeon using the conventional technique, without disadvantages regarding surgical time.

## 2. Materials and Methods

### 2.1. Study Population

**Figure 1:**
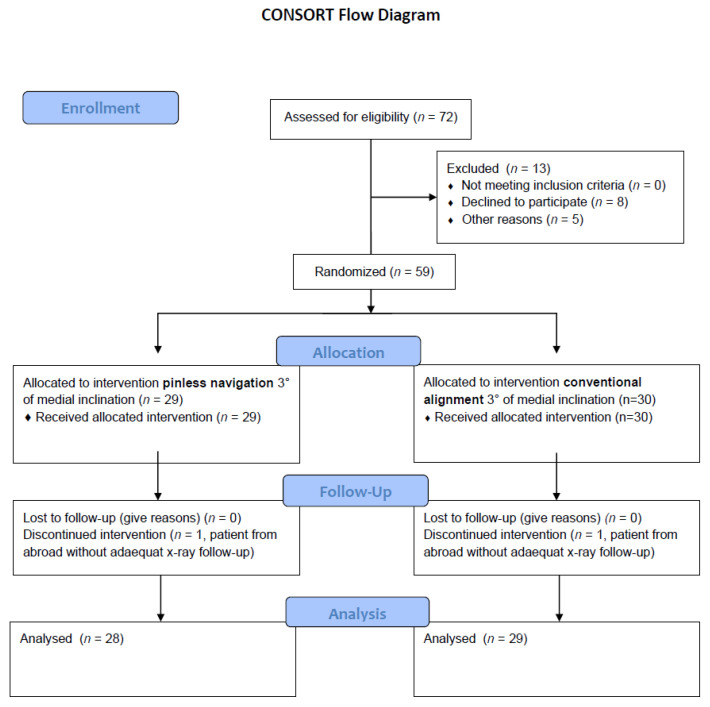
Flow diagram illustrating patient enrolment, allocation, follow-up and analysis.

The study was approved by the local Ethics Committee (No. 19-1548-101) and written informed consent was given by all patients participitating in this study. The trial has been notifed at the German Clinical Trials Register (ID DRKS00025189). The inclusion criteria in the study, for the consecutive series of 30 patients with unicompartmental osteoarthritis of the knee eligible for unicompartmental arthroplasty, were that patients were aged between 40 and 80 years, exhibited intact cruciate and collateral ligaments, had no narrowing of the contralateral joint space, had no evidence of higher grade patellofemoral disorders or arthritis and an overall operational capability. Exclusion criteria were symptomatic retropatellar or contralateral arthritis, fixed deformity > 15°, inflammatory joint diseases, status after ligamentary reconstruction (e.g., ACL-reconstruction) and status after osteotomy. The characteristics of the study population are shown in Table 1. A treatment group and a control group were formed, each with 30 patients. The treatment group was operated on using the imageless navigation technique, and the control group using the conventional surgical technique, in each case with a target value of 3° varus of the tibial component. Two patients, one in each group, denied further participation in the study. In addition, one patient was indicated for a lateral UKA. This case was excluded after surgery as it would be misleading for the homogeneity of the study. Therefore, a total of 57 patients were considered for radiographic analysis. Figure 1 shows a flow diagram illustrating patient enrolment, allocation, follow-up and analysis.

### 2.2. Surgical Treatment

All procedures were performed between October 2019 and June 2020 by the same experienced senior surgeon (>200 knee UKA/year). The patient was placed in a supine position under spinal anesthesia and a tourniquet was inflated with 100 mmHg above the systolic blood pressure at the time of inflation. The quad-sparing-approach was performed in all patients. Subjects received an implant with a fixed bearing platform (Persona Partial Knee, Zimmer Biomet, Warsaw, IN, USA) for medial arthritis in 59 cases and a Physica ZUK (Lima Corporate, UD, Italy) in one case for lateral arthritis target. However, due to a different surgical approach and for the purpose of homogeneity in the evaluation, this case was removed for the data analysis. All cases were fully cemented and the same target values and radiographic analysis were used. The target component was the tibial implant, which was to be placed in 3° varus. In order to achieve that, a computer-assisted, pinless navigation device (Knee 2.6, Brainlab AG, Feldkirchen, Germany) was used for 30 patients. The accuracy of this system has already been investigated in several studies and was verified to be a precise and reliable pinless navigation system [7,8,9,10]. The setup consisted of a stand-alone computer with infrared cameras, controlled by a touch screen, and a pointer with reflector spheres as well as a reflector array. After bony exposure, the anatomical landmarks were mapped by the pointer, in particular the medial and lateral malleolus, the tibial anatomic axis and a.p.—direction of the tibia. Subsequently a standard extramedullary alignment rod from the Zimmer/Biomet system was attached perpendicular to the tibia and the reflector array was inserted into the cutting slot. Next, the correct alignment of the tibial cut was verified by the navigation system. For the remaining 30 patients, the standard extramedullary alignment rod was used to align the tibial component at 3° varus without computer-assisted guidance.

**Figure 2:**
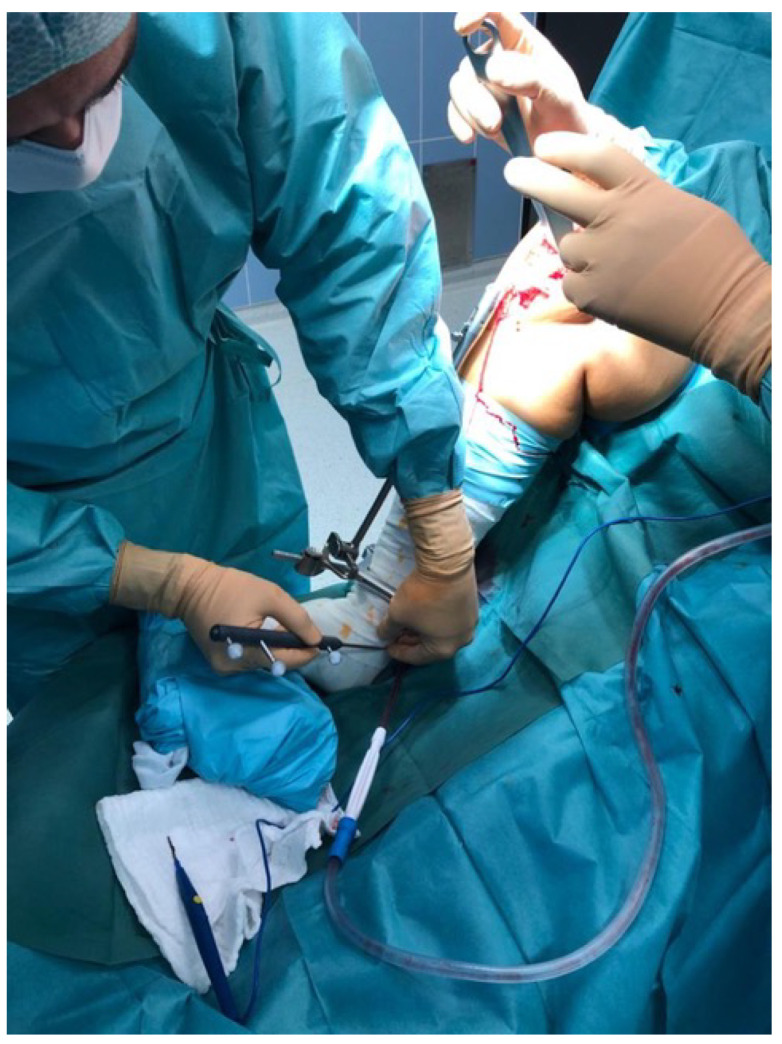
The surgical setup is shown. Here, the surgeon was referencing the medial malleolus. Light-reflecting spheres, attached to the pointer, were detected by an infrared camera positioned in the background.

**Figure 3:**
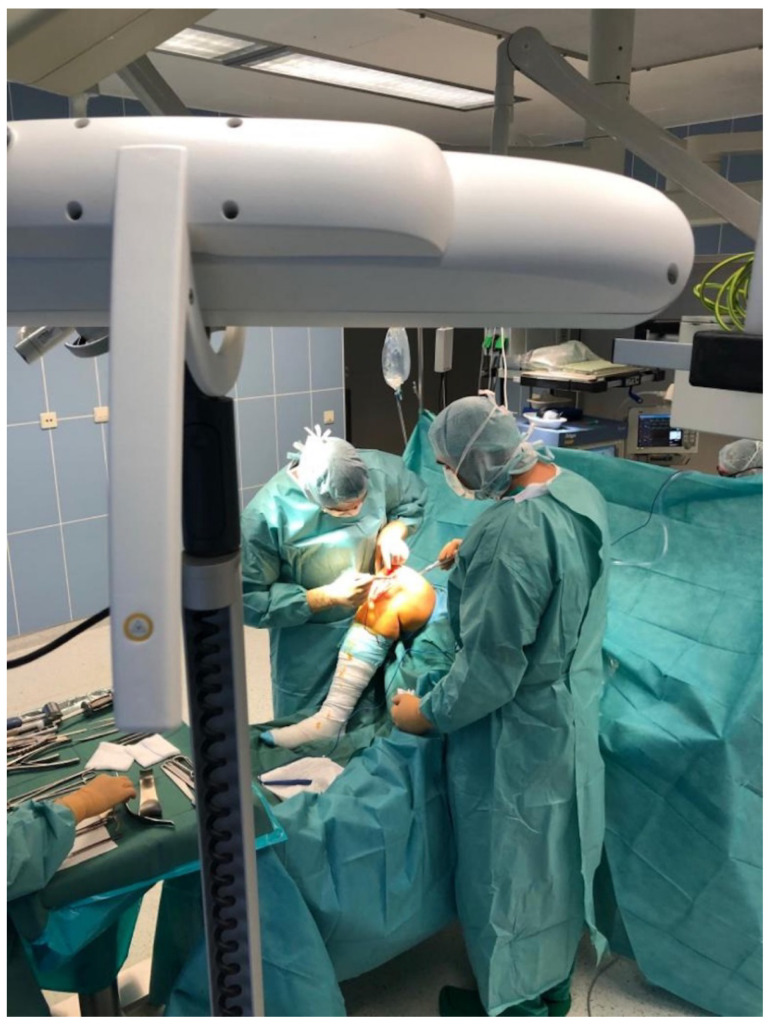
Shows the infrared camera facing the surgical field for detecting the light-reflecting spheres of the pointer.

Figure 2 shows the surgical setup. Here, the surgeon was referencing the medial malleolus. Light-reflecting spheres are attached to the pointer, which are detected by the infrared camera positioned in the background. Note that no pins needed to be drilled into the bone. Figure 3 shows the infrared camera facing the surgical field for detecting the above mentioned, light-reflecting spheres.

### 2.3. Radiographic Analysis and Clinical Follow up

**Figure 4:**
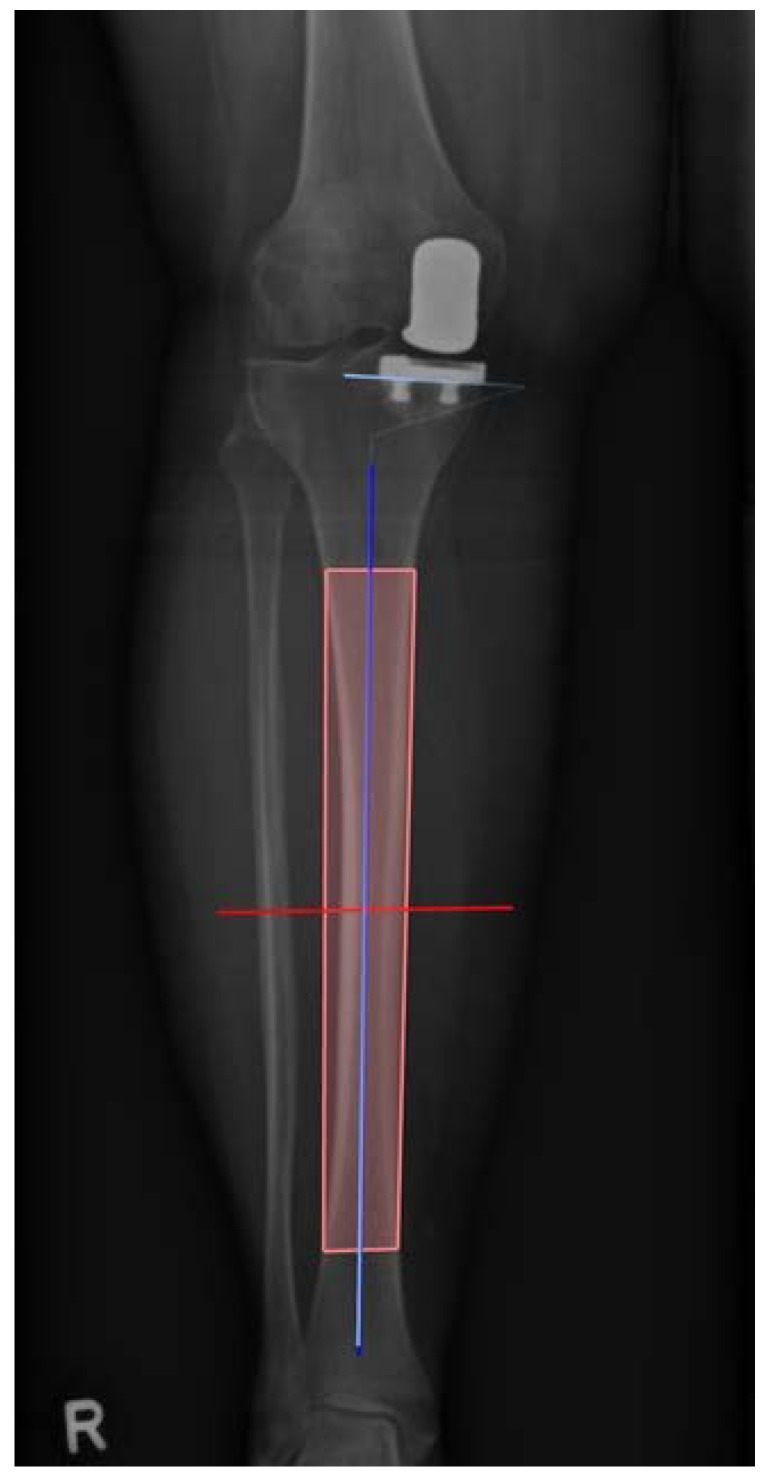
Illustration of the measurement process. Two points on the medial and two on the lateral cortex were marked and thus the longitudinal axis was calculated. Next a tangent was drawn to the lower border of the tibial component and thus the medial inclination calculated between tangent and longitudinal axis. In this case of group 1 the angle was 87.4° varus (R = right side).

Within one year postoperatively, the clinical and radiological follow-up of the patients took place. Patients were routinely examined and surgery sites were controlled. Standard weight bearing radiographs in lateral projection as well as long-leg radiographs were taken. Subsequently, the inclination of the tibial component was determined. Radiographic analysis was performed in Horos for Mac, Version 3.3.6 [11]. First, the anatomical axis of the tibia was determined in the a.p. long-leg radiographs according to Paley [12]. Four points of the two cortices were marked, two in the proximal and two in the distal metaphysis, each with one on the lateral and one on the medial corticalis, resulting in a rectangle shape. Based on this, the software automatically calculated the center of the four landmarks and thus the longitudinal axis. Following that, the angle of the tibia component was calculated from the previously determined axis and the lower edge of the tibial implant. Figure 4 represents the measurement process. Two points on the medial and two on the lateral cortex were marked and thus the longitudinal axis was calculated. Next a tangent was drawn to the lower border of the tibial component and thus the medial inclination calculated between tangent and longitudinal axis.

**Figure 5:**
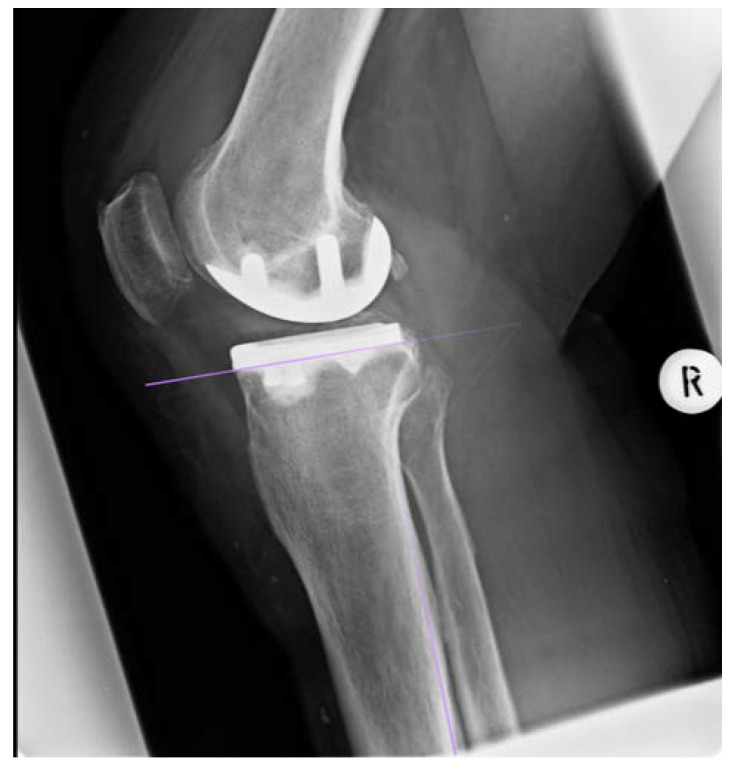
Illustration of the measurement process. For determining the posterior slope, a tangent was drawn next to the posterior cortical axis, another one next to the lower border of the tibial component. In this case the posterior inclination was 87.5°.

The posterior inclination of the tibial component was determined from the lateral radiographs according to Faschingbauer et al. [13]. One tangent was applied to the posterior cortex, another to the caudal end of the component. Thus, the angle was calculated between the two lines. This process of measurement is shown in Figure 5.

### 2.4. Statistical Analysis

For statistical analysis, continuous data are presented as means and standard error of the mean. Group comparisons were performed by two-sided t-tests for independent variables. Absolute and relative frequencies were given for categorical data. Differences of *p* < 0.05 were considered statistically significant. IBM SPSS Statistics 26 (SPSS Inc, Chicago, IL, USA) was used for analysis. Post hoc power analysis was performed for the navigation data. The analysis in G*Power 3.1 for Mac resulted in a calculated sample size of 28 subjects for both groups to achieve a statistical power of 1-beta = 0.9 and an alpha of 0.05.

## 3. Results

Within one year postoperatively, no surgery site infections were noted, all wounds healed per primam and no revision was necessary. No dropouts because of failure of the navigation or intraoperative decision to switch to TKA were noted.

In the treatment group, the mean position of the tibial component was 87.6° +/− 1.0°. Minimum and maximum medial inclination was 86.1° and 89.7°, respectively, resulting in a range of 3.6°. The 25th percentile was 86.9° and the 75th percentile was 88.3°. The mean posterior inclination of the tibial component was 86.8° +/− 1.9°. Minimum and maximum posterior inclination was 84.0° and 90.0°, resulting in a range of 6°.

In the control group, the mean position of the tibial component was 87.3° +/− 1.2°. Minimum and maximum medial inclination was 84.1° and 89.3°, respectively, resulting in a range of 5.2°. The difference between the two groups was not significant, *p* = 0.3. The mean posterior inclination of the tibial component was 86.9° +/− 1.6°. Minimum and maximum posterior inclination was 83.9° and 89.7°, resulting in a range of 5.8°.

**Figure 6:**
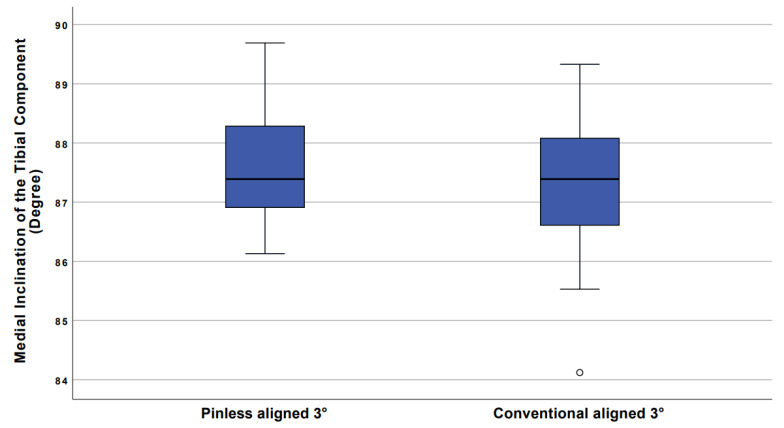
Mean position of the tibial component in coronar plane. Boxplots show range of medial inclination for navigation and conventional group. With the navigation technique, outliers could be reduced as well as range of medial inclination. One outlier in the conventional aligned group was marked with “o”.

Figure 6 presents boxplots, which show the range of medial inclination for navigation and conventional groups.

**Figure 7:**
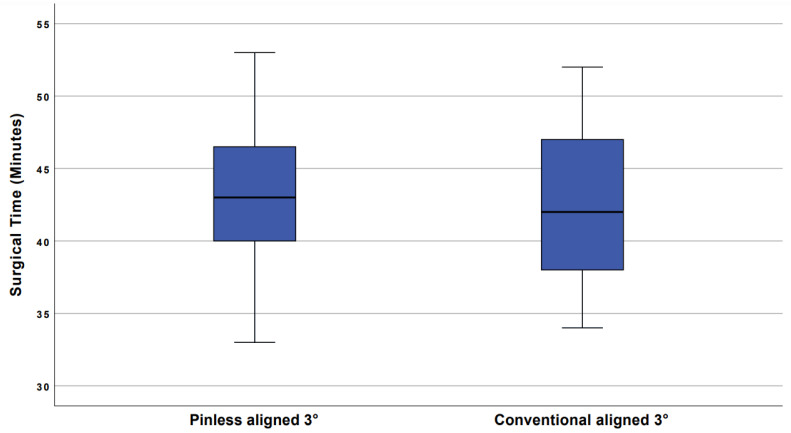
Mean incision to suture time. Boxplots show range of incision to suture time. In the navigation technique, the range and mean time was slightly higher. One outlier with 62 min in the navigation group was noticed.

Mean incision to suture time in the navigation group was 44.2 min +/− 4.4 and 42.7 min +/− 5.1 in the control group. The difference was statistically not significant, p = 0.13. Boxplots showing the range of incision to suture time are illustrated in Figure 7.

## 4. Discussion

The accuracy of the implantation and the correct three-dimensional alignment of the unicondylar knee replacement are essentially responsible for the clinical outcome. This requires a considerable amount of extensive clinical experience by the surgeon to achieve the required precision. Registry data show that experienced surgeons increase the survivorship of unicompartmental knee arthroplasty in contrast to surgeons who present only a low annual volume of UKA [14].

In the present study, we demonstrated that pinless navigation can achieve equivalent implantation accuracy like an experienced surgeon. Furthermore, the number of outliers could be reduced compared to an experienced surgeon. The previously documented minimum number of operations per surgeon per year for a good surgical result could thus be significantly lower when a navigation technique is used for implantation. A previously study defined a threshold of 12 UKAs per year. Below this, surgeons cause a revision rate twice as high as surgeons with more than 12 implantations per year. However, this is only valid for the conventional alignment technique [15].

Additionally, the formerly documented proportion of 40–60% unicondylar prostheses in the number of surgeries performed annually by a single surgeon, as a prerequisite for a satisfying outcome, could; therefore, be re-discussed [16].

It can be assumed that the deviation in three-dimensional alignment, which often results when conventional instruments are used, is the cause of the increased failure rate. The rationale for desired positioning of a unicondylar partial knee prosthesis is that the alignment should be according to the anatomy, since the lateral and patellofemoral portions are not replaced.

In recent years, this consideration has led to the belief that a slight varus alignment provides better results because it is more consistent with anatomic conditions [4]. Although the optimal positioning is still unclear, observations show that, on the one hand, excellent results can be achieved with this technique, but the degree of maximum deviation is even lower. Thus, a deviation of 5° from the mechanical tibial axis appears to be the threshold for a then strongly increasing probability of loosening and a worse outcome [17].

So far, it has been shown that computer-assisted navigation could implant UKAs as accurately or even more accurately in terms of clinical outcome than when using the conventional technique [18,19]. The application of pins has several disadvantages as there is the risk of fracture development, the possibility of local infection, and additional damage to the soft tissue [20,21]. Moreover, a lower amount of additional time was involved [22].

To the best of our knowledge, the presented study is the first investigating pinless navigation in UKA. The results showed that, with this type of navigation, even an experienced surgeon could achieve target values with lower outliers regarding component placement than with the conventional technique. The range for pinless navigation was 3.6°, which was significantly less than the range of 5.2° for conventional alignment technique.

The fact that pinless navigation can reduce outliers compared to conventional implantation has already been shown in total knee arthroplasty [7,10]. In the study presented here, outliers could also be avoided with the navigated technique in comparison to conventional alignment technique, with which also one outlier of 84.1° was produced, even in a high volume surgeon.

The reduced range of the degree of medial inclination of the tibial component compensates for the minimally extended incision to suture time. It was 43.2 min for the implantation with the pinless navigation and 42.7 min without navigation.

In addition, our data showed an increased range of operation times in pinless navigation compared to conventional instrumentation. This could be due to the fact that the use of the navigation unit can cause delays for technical reasons (e.g., registration of landmarks).

Our study has several limitations. First, radiographs were taken from a clinical routine. These sufficiently met the clinical requirements, but for scientific purposes, it must be mentioned that sometimes the prosthesis was not completely orthograde by the central beam, so that there was a certain tilting, which made the measurement more difficult.

Second, all operations and measurements were performed by one single surgeon, so that no intra- or inter-rater reliability values were available.

Third, the determination of the position of the tibial component was performed after cementing, consequently a different thickness of the cement mantle may have influenced the result.

## 5. Conclusions

For the first time, it could be shown that pinless navigation can significantly reduce the percentage of outliers with similar implantation accuracy. The clinical relevance is that even an inexperienced surgeon can achieve accuracy comparable to that of a highly experienced surgeon using pinless navigation. The disadvantages of pin-based navigation can; thus, be completely avoided. The extension of the operation time can be estimated as not relevant.

Besides the advantages, of course, the learning curve and the acquisition costs must be considered.

## Figures and Tables

**Table 1:** Patient characteristics.

	Treatment	Control
n	28	29
Gender (male/female)	16/12	14/15
Age (years)	64.0	63.4
Treatment side (left/right)	10/18	14/15

## Data Availability

Data sharing is not applicable to this article.
